# High rates of deferring antiretroviral treatment for patients with HIV and substance use disorders: Results from a national sample of HIV physicians in Ukraine

**DOI:** 10.1371/journal.pone.0305086

**Published:** 2024-07-19

**Authors:** Taylor D. Ottesen, Jeffrey A. Wickersham, Juliana C. Lawrence, Sergii Antoniak, Oleksandr Zezuilin, Maxim Polonsky, Svitlana Antonyak, Julia Rozanova, Sergii Dvoriak, Iryna Pykalo, Myroslava Filippovych, Fredrick L. Altice

**Affiliations:** 1 Department of Internal Medicine, Section of Infectious Diseases, AIDS Program, Yale School of Medicine, New Haven, Connecticut, United States of America; 2 Harvard Combined Orthopaedic Residency Program, Boston, MA, United States of America; 3 L.V. Gromashevsky Institute of Epidemiology and Infectious Diseases of the National Academy of Medical Science of Ukraine, Kyiv, Ukraine; 4 Ukrainian Institute on Public Health Policy, Kyiv, Ukraine; 5 Keck Graduate Institute, Claremont, CA, United States of America; 6 Department of Epidemiology of Microbial Diseases, Yale University School of Public Health, New Haven, Connecticut, United States of America; 7 Centre of Excellence in Research on AIDS (CERiA), University of Malaya, Kuala Lumpur, Malaysia; The Technical University of Kenya, KENYA

## Abstract

**Background:**

HIV incidence and mortality are increasing in Ukraine despite their reductions globally, in part due to suboptimal antiretroviral therapy (ART) coverage in key populations of people with HIV (PWH) where the epidemic is concentrated. As physicians are gatekeepers to ART prescription, stigma and discrimination barriers are understudied as a key to meeting HIV treatment targets in key populations.

**Methods:**

A national sample (N = 204) of ART-prescribing physicians in Ukraine were surveyed between August and November 2019. Participants underwent a series of randomized, hypothetical HIV clinical scenarios and decided whether to initiate or defer (or withhold) ART. Scenarios varied based on 5 distinct CD4 counts (CD4: 17, 176, 305, 470, or 520 cells/mL) and 10 different PWH key populations. *Z* scores and McNemar’s test for paired samples were used to assess differences between key populations and CD4 count. Feeling thermometers were used to assess stigma-related measures toward key populations among physicians.

**Results:**

Physicians were highly experienced (mean = 19 years) HIV treaters, female (80.4%), and trained in infectious diseases (76.5%). Patients who drink alcohol (range: 21.6%-23.5%) or use (PWUD range: 16.7%-20.1%) or inject (PWID range: 15.5%-20.1%) drugs were most likely to have ART deferred, even at AIDS-defining CD4 counts. PWID maintained on methadone, however, were significantly (p<0.001) less likely to have ART deferred compared with those who were not (range: 7.8%-12.7%) on methadone. Men who have sex with men (range: 5.4%-10.8%), transgender women (range: 4.9%-11.3%), sex workers (range: 3.9%-10.3%),and having an HIV-uninfected sex partner (range: 3.9%-9.3%) had the lowest likelihood of ART deferral. Increasing levels of stigma (i.e., feeling thermometers) towards a key population was correlated with ART deferral (i.e., discrimination).

**Conclusions:**

Despite international and Ukrainian guidelines recommending ART prescription for all PWH, irrespective of risk or CD4 count, ART deferral by experienced HIV experts remains high in certain key populations, especially in PWH and substance use disorders. Strategies that initiate ART immediately after diagnosis (i.e., rapid start antiretroviral therapy), independent of risk group, should be prioritized to truly mitigate the current epidemic.

## Introduction

Before scaling up antiretroviral therapy (ART) and strategies that focused on HIV treatment as prevention as part of the UNAIDS 90-90-90 strategy, HIV incidence and mortality steadily increased [1]. As new data emerged, international agencies transitioned to a policy to treat people with HIV (PWH), irrespective of CD4 count, primarily as early treatment improves both individual and public health through treatment as prevention [2]. Consequently, HIV incidence and mortality markedly decreased globally by 2015 [3], yet did not where ART coverage was limited, especially in key populations. The largest region to have failed to reduce HIV incidence and mortality during this period is Eastern Europe and Central Asia (EECA), a region where HIV is concentrated in key populations, especially people who inject drugs (PWID) and their sexual partners [3]. These disparities persist through 2021 [4].

The World Health Organization (WHO) modified its HIV prevention and treatment guidelines in 2015 to initiate ART, regardless of CD4 cell count, as soon as possible after diagnosis. Previously ART had been limited to PWH with CD4< 500 cells/mL, with special priority given to those with CD4<350 cells/mL [5]. Ukraine adopted these guidelines in 2016. By year-end 2019, ART was prescribed only to 98,237 (40%) of the estimated 240,000 PWH in Ukraine [6], substantially lower than recommended by the 90-90-90 strategy needed to reduce HIV incidence by 2030 [7].

Multi-level factors in the sociological model may influence ART scale-up, yet physicians in healthcare settings are the major gatekeepers to ART access and can substantially influence to scale-up efforts. Several studies have suggested that stigma toward key populations in Ukraine is high, including toward PWID, men who have sex with men (MSM), transgender women (TGW) and female sex workers (FSW) [8, 9], which may translate to healthcare providers.

Studies elsewhere suggest that some physicians’ prescribing practices may limit ART acquisition to key risk populations, effectively undermining important treatment and prevention strategies [10–13]. Other studies show that healthcare providers hold prejudice against PWH [14, 15], including medical students, the future prescribers of medications, which may lead to downstream discrimination in the healthcare system [13, 16, 17]. What remains unknown is whether these same prejudices may be held by healthcare providers who provide direct care and life-saving medications to PWH, and to what extent they extend to key populations, and influence critical HIV intervention in high-risk regions like Ukraine.

Although other studies have examined the intention to discriminate by healthcare against key populations outside EECA, this study is the first to investigate physician’s decision to defer (or delay or withhold) ART among key populations in a region that is experiencing an increase in new HIV cases while the rest of the world has decreasing incidence. To do so, we surveyed a convenience sample of 204 practicing physicians across the country who treat HIV patients in Ukraine to investigate: 1) potential implicit biases and stigmas surrounding PWH with a focus on key populations; 2) if those stigmas affect how physicians provide access to ART in these key populations, and if so; 3) are any factors that may lead physicians in this region to be less likely to espouse these stigmas as has been shown elsewhere [18–20].

## Methods

### Survey population

Between August 1^st^ and November 9^th^ 2019, a national sample of registered ART-prescribing physicians registered with the Ukrainian Center for Public Health were contacted by researchers at the Ukrainian Institute on Public Health Policy (UIPHP). Overall, 346 registered HIV-treating clinicians were emailed an invitation with a link to the survey, which was designated as anonymous, voluntary, and the option to submit their name into an unlinked lottery for a tablet computer at the end of the survey. Of these, 315 gave written consent to participate in the survey with 84 (26.7%) unable to participate because they did not inclusion criteria of having prescribed ART within the last 12-months or they were not a licensed physician who could prescribe medications. Of the 231 eligible providers who completed the survey, 27 (11.7%) had a high non-response to key questions including the independent variable, resulting in 204 (88.3%) participants whose data were included in the analysis.

### Instruments

The online survey was conducted using Qualtrics and modified from two previous surveys conducted with ART-prescribing physicians in the U.S. and Malaysia [17, 19]. Survey questions were expanded to include other key populations and adapted for the Ukrainian context. The final survey was translated and back-translated into Russian and Ukrainian to ensure cultural competency [21]. The survey was further piloted with 20 Ukrainian ART-prescribing physicians and some minor adjustment of wording of the survey questions was performed to ensure question comprehension. Participants were allowed to complete the final survey in either English, Ukrainian, or Russian. Demographic and medical experience information was collected in addition to responses to clinical scenarios (described below).

### Clinical scenarios

Five different clinical scenarios were presented to participants and each scenario had a different CD4 lymphocyte count (CD4 17, 176, 305, 470, or 520 cells/mL). Within each scenario, 10 patient situations were presented. Nine of these situations consisted of key populations or associated circumstances and 1 non-key population situation was used as a control (a patient with HIV acquired through heterosexual contact). The result was 50 unique clinical situations. Definitions of key populations were based on guidelines outlined by the WHO [22]. Participants were asked whether they would initiate ART “now” or defer ART for “later” for each of the 50 clinical situations. Participants were to mark initiate ART “now” to indicate their plan to begin ART immediately or defer it for later for each patient in a specified clinical scenario. Deferring ART was used as discrimination construct as it deviates markedly from national and international recommendations.

For each clinical scenario, participants were told the presenting patient is currently infected with HIV but entirely asymptomatic, has no evidence of opportunistic infections or tuberculosis, and is highly interested in beginning ART now. The clinical scenarios were presented randomly in order not to guide choice selection based on social desirability. The four different CD4 thresholds were chosen to reflect patient prototypes that reflected prior ART guidelines for treatment that were consistent with successive changes in recommendations for ART initiation (<200 cells/mL, <350 cells/mL, <500 cells/mL and universal treatment). The recommendation at the time of the survey was to initiate ART at the time of diagnosis irrespective of CD4 count. A fifth scenario, CD4 = 17 cells/mL (i.e., CD4<50), was added to reflect patients with very advanced HIV disease who without ART, would be expected to have an extraordinarily high risk for opportunistic infection or death in the next six months. For the final analyses, we focused on PWH with CD4 counts <200 cells/mL. Deferral of ART was highest for those with less advanced CD4 and analyzing data for other CD4 strata did not substantially impact the findings.

### Stigma-related measures

To further explore attitudes of providers towards patients with HIV, particularly the most at-risk key populations in Ukraine, several survey items were included as has been done in prior studies [20]. Specifically, feeling thermometers were utilized to delineate provider’s attitudes (stereotypes) towards general medical patients without HIV in relation to those with HIV and ongoing alcohol and drug use [PWID, people who use drugs (PWUD), or person with alcohol use disorder (AUD)], those with social vulnerability (recently in prison, living alone, or who acquired HIV via heterosexual sex), and sexual and gender minorities (female sex worker, has HIV- sex partner, MSM, and transgender women). PWID who were engaged in addiction treatment, reflected by being on opioid agonist therapies (OAT) like methadone or buprenorphine, were also included. Using a 100-point scale, the feeling thermometers asked physicians to indicate their general feelings towards each group, with 1 designated as very negative and 100 designated as very positive. Discrimination intent and HIV-related stigma is expected to be inversely correlated with this score, with less-favorable attitudes toward a group resulting in a smaller score, thus indicating larger negative bias toward them. Feeling thermometers have been previously established as dependable tools to measure prejudice [23] and have previously been used to assess attitudes of physicians [20], medical students [16, 24], and healthcare providers in other countries [20]. Other stigma-related constructs were measured using a validated 17-item HIV stigma scale [25], with subscales measuring prejudice, internalized shame, fear, stereotypes and discrimination intent using the HIV Stigma Framework adapted for PWID [26].

### Other variables and definitions

Location of clinic was stratified as urban, suburban or rural using Ukrainian national statistics based on where the participant selected their practice site. The U.S. President’s Emergency Plan for AIDS Relief (PEPFAR) provides substantial financial support HIV services in regions most impacted by HIV, which we stratified as high and medium burden based on the number of PWH who reside in that settings. All other regions do not qualify for PEPFAR support.

### Data analysis

Analyses were conducted using SPSS Version 26. Descriptive statistics were reported using mean and standard deviations. *Z* scores and McNemar’s test for paired samples of control patients and high-risk populations were used to assess differences between key-patient prototypes and CD4 counts. AIDS-defining CD4 thresholds were defined as CD4<200 (i.e., 176 and 17) while non-AIDS-defining thresholds were defined as >200 (i.e., 520, 470, and 305). As there were no differences in high and medium burden settings, PEPFAR was dichotomized into PEPFAR and non-PEPFAR regions. Bivariate logistic regressions were used to examine associations between demographic characteristics including the 12 regions prioritized by PEPFAR, clinical factors, and stigma-related constructs on physician’s decision to defer ART for PWH with a CD4 of 305 cells/mL. Candidate covariates for the multivariable model were evaluated for multicollinearity using a variance inflation factor <7 and a tolerance threshold of <0.20. All covariates identified as statistically significant in bivariate analyses at *p*<0.05 were included in the multivariable logistic regression model. In the multivariable model, we regressed the dependent variable onto the covariates using simultaneous entry.

This study received exemption by Yale’s Human Investigations Committee as well as the Ukrainian Institute on Public Health Policy. Consent to participate and Consent to publish Informed consent was obtained from all individual participants included in the study. Each gave written consent for their participation. No minors were included in the study. Further, the authors affirm that human research participants provided informed consent for publication of their aggregated data in verbal and graphical form.

## Results

### Demographics

Demographic characteristics of study participants are similar to practicing HIV physicians in Ukraine, being mostly women (77.9%), practicing at an AIDS center (53.4%), in urban settings (95.6%), in either a high or medium burden PEPFAR region (72.5%) (Table 1). Mean years of clinical practice was about 20 years, with none being in practice <5 years; and most were infectious disease specialists (77.5%). Overall, their clinical practices included, on average, over three-quarters (77.3%) of their patients being PWH. Further, the majority of their professional time involved direct patient care (71.1%) with the majority of this patient care time being HIV care (75.5%) and they saw an average of 79.4 patients with HIV per week.

**Table 1:** Demographic and clinical characteristics of HIV physicians (N = 204).

Variable	
Mean Age, Years (*SD*)	45.2 (10.7)
Mean Years practicing medicine (*SD*)	20.4 (10.2)
Sex	
Female, n (%)	159 (77.9)
Male, n (%)	45 (22.1)
Type of HIV clinical setting	
AIDS center, n (%)	109 (53.4)
Multi-specialty center or hospital, n (%)	60 (29.4)
Primary care or family medicine, n (%)	3 (1.5)
Other, n (%)	32 (15.7)
Location of HIV clinical setting	
Urban, n (%)	195 (95.6)
Suburban, n (%)	3 (1.5)
Rural, n (%)	6 (2.9)
HIV Practice Location within PEPFAR Region	
Yes–high burden, n (%)	98 (48.0)
Yes–medium burden, n (%)	50 (24.5)
No, n (%)	56 (27.5)
Specialization	
Infectious diseases, n (%)	158 (77.5)
Pediatrics, n (%)	11 (5.4)
Therapist, n (%)	7 (3.4)
Obstetrics and gynecology, n (%)	5 (2.5)
Other, n (%)	23 (11.3)
Mean Percent of professional time in patient care (*SD*)	71.1 (22.5)
Mean Percent of patient care devoted to HIV care (*SD*)	75.5 (27.5)
Average weekly patient load (*SD*)	79.4 (92.2)

*M* = mean; *SD* = standard deviation; PEPFAR = U.S. President Emergency Plan for AIDS Relief

*n = 185

### Stigma-related measures

**Fig 1:**
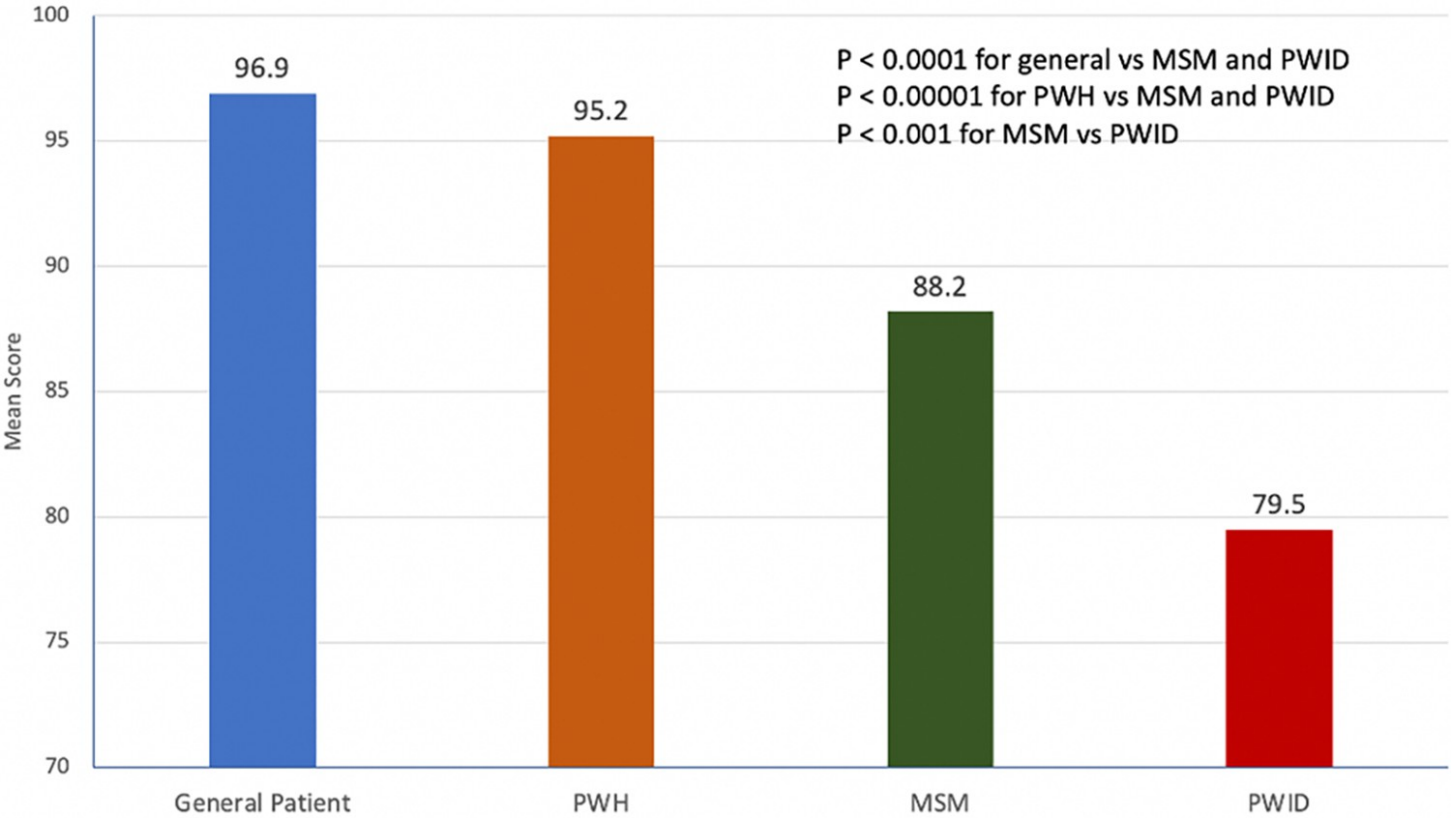
Mean feeling thermometer scores toward various populations of people with or at risk for HIV (N = 204). PWH = person with HIV; MSM = men who have sex with men; PWID = person who injects drugs.

Fig 1 displays the mean feeling thermometer scores for a general patient, relative to other vulnerable populations including PWH, MSM and PWID. Mean scores were lowest (more negative attitude) for PWID (M = 79.5) relative to both general patients (M = 96.9) or PWH (M = 95.2). Relative to general medical patients, there was no statistically different mean score toward PWH. The MSM and PWID groups, however, had significantly lower scores relative to both of these groups. The difference in scores between MSM (M = 88.2) and PWID (M = 79.5) was also significant, with physicians having the lowest score toward PWID.

### Deferral of ART

**Fig 2:**
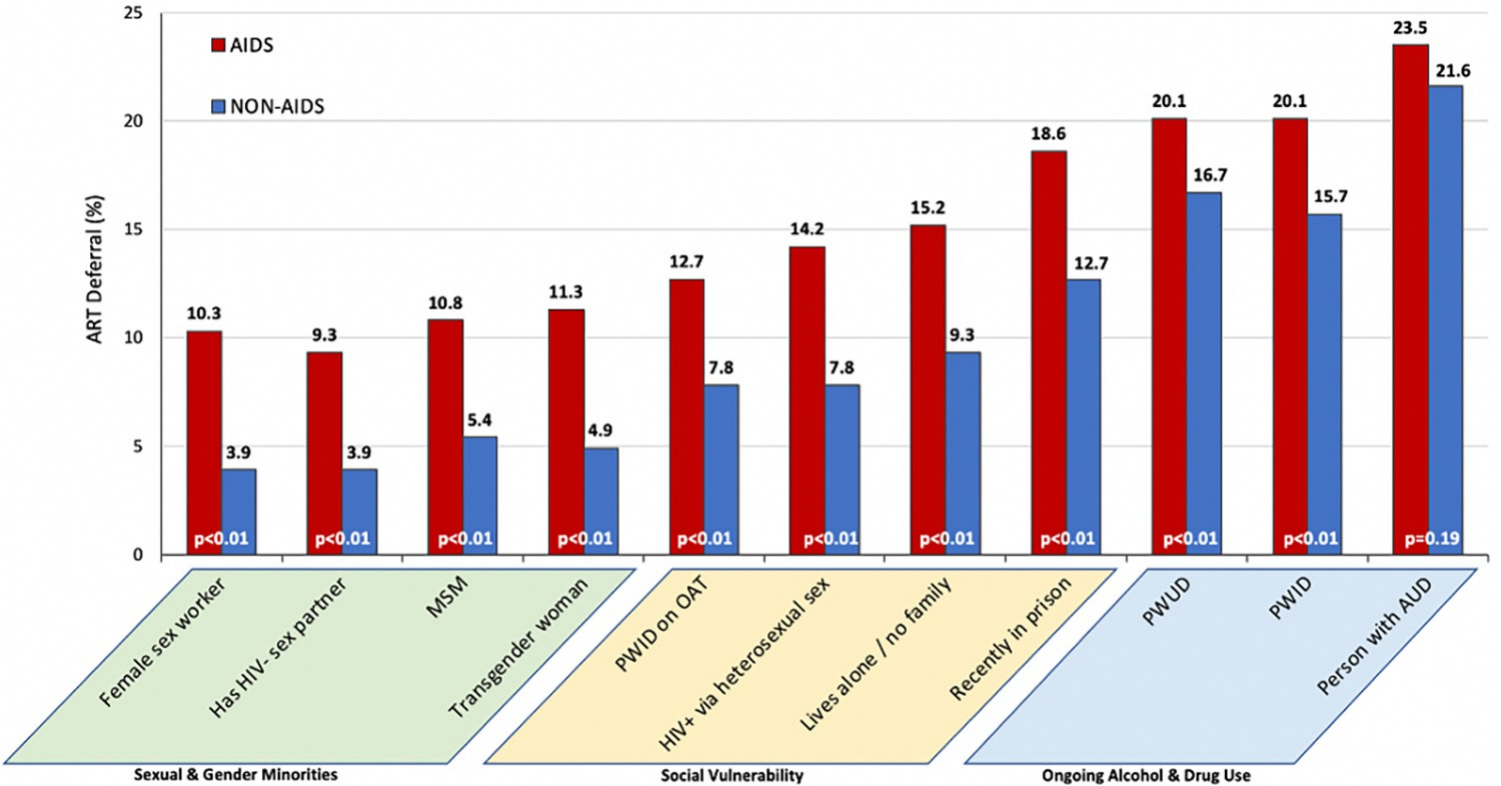
Proportion of HIV physicians who would defer antiretroviral therapy for key populations, stratified by HIV disease severity (N = 204). *NOTE*: AIDS defining diagnosis was defined as CD4+ count of 17 or 176 while non-AIDS defining diagnosis were defined as CD4+ count of 305 or 470.

Fig 2 shows the extent, expressed as a proportion, to which physicians would defer or withhold ART for each key population for patients interested in starting ART immediately. One key population who ART would be deferred by physicians was those with an alcohol use disorder (23.5% of physicians would defer if the patient met AIDS-defining criteria and 21.6% if they did not). Irrespective of which key populations studied, physicians would defer ART more often for those with a CD4 count consistent with AIDS-defining diagnosis (<200 cells/mL) relative to those with higher CD4 counts. While deferral levels of ART for PWUD and PWID was similarly high, if either of these groups were prescribed OAT, the likelihood of deferral was significantly lower (12.7% and 7.8% for AIDS and non-AIDS patients, respectively) relative to those not prescribed OAT. The proportion of prescribers who would defer ART the least included patients with a sex partner without HIV (thus at risk for transmitting HIV to their sexual partner) and female sex workers; ART deferral was 9.3% and 10.3% for AIDS patients in both groups, respectively, and 3.9% of non-AIDS for both groups.

Of interest is the finding that a high proportion of physicians would withhold ART in people using drugs and those recently released from prison, relative to most other groups. While stigma toward PWID, measured as low scores on feeling thermometers and negative stereotypes toward PWID, in part, explain this finding. Unmeasured factors for this reluctance to prescribe ART to these groups, however, might include inaccurate perceptions that these groups may be poorly adherent to medications (unlike findings in systematic reviews and meta-analyses) [27]. Alternatively, stated withholding of ART could be due to concerns about impending incarceration, which was observed in one study in Malaysia [19]. Such perceptions, however, are not aligned with the evidence.

In the multivariate analysis of PWID, no sociodemographic or clinical practice characteristics were associated with ART deferral (Table 2). If PWID were on OAT, however, the likelihood of deferring ART was reduced by 97% (AOR = 0.03; 95%CI = 0.01–0.13) but was increased 2.4-fold when physicians expressed high levels of negative stereotypes toward PWID (AOR = 2.41; 95%CI = 1.18–5.72). There were no differences in deferral patterns between physicians practicing in PEPFAR versus non-PEPFAR regions (extra financial resources are available in PEPFAR regions). Further, when examining stigma-related constructs, prejudice, internalized shame, fear, stereotype, and discrimination intent toward PWID all significantly elevated the odds of ART deferral by physicians.

**Table 2:** Bivariate and multivariable correlates of physician decision to defer initiation of antiretroviral therapy among people who inject drugs* (N = 204).

	OR	95%CI	*p*	aOR	95%CI	*p*
**Sociodemographic factors**						
Male sex	0.47	0.21–1.07	0.072			
Age (years)						
18–35	Ref	-	-			
36–50	1.32	0.50–3.46	0.574			
51 and older	0.68	0.23–2.02	0.486			
**Clinical factors**						
Years practicing medicine						
0–10 years	Ref	-	-			
11–20 years	1.42	0.46–4.40	0.542			
21–30 years	0.89	0.28–2.78	0.839			
31 or more years	0.80	0.21–3.10	0.747			
Infectious disease specialist	1.05	0.42–2.60	0.921			
Prescribes methadone	0.32	0.04–2.46	0.270			
Prescribes buprenorphine	1.37	0.28–6.75	0.702			
Percent of time in patient care						
0–50%	1.29	0.55–3.02	0.555			
51–100%	Ref	-	-			
Percent of time in HIV care						
0–50%	0.99	0.40–2.46	0.978			
51–100%	Ref	-	-			
Works in PEPFAR region	1.42	0.46–2.33	0.443			
Would defer ART						
PWID	Ref					
PWID on OAT	0.04	0.012–0.135	**<0.001**	0.03	0.01–0.13	**<0.001**
**Stigma**						
Feeling thermometers						
PWH thermometer	0.99	0.97–1.03	0.846			
PWID thermometer	0.98	0.96–0.99	**0.004**			
MSM thermometer	0.99	0.98–1.01	0.533			
Stigma-related constructs						
PWID prejudice	1.93	1.11–3.34	**0.019**			
PWID internalized shame	1.95	1.21–3.16	**0.006**	1.33	0.60–2.95	0.479
PWID fear	1.76	1.09–2.85	**0.021**	0.86	0.42–1.77	0.479
PWID stereotype	2.78	1.43–5.38	**0.002**	2.41	1.18–5.72	**0.018**
PWID discrimination intent	2.27	1.30–3.97	**0.004**	1.84	0.90–3.76	0.094

PWH = people with HIV; PWID = people who inject drugs; MSM = men who have sex with men

*model restricted to scenario of PWID with CD4 lymphocyte count = 305 cells/mL

**Fig 3:**
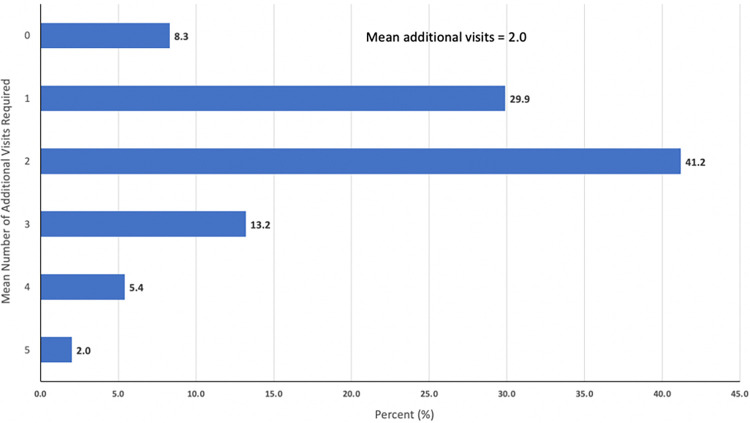
Number of additional clinic visits required by physician prior to initiating antiretroviral therapy (N = 204).

Despite international and Ukrainian guidelines recommending ART initiation in PWH as soon as possible after diagnosis and regardless of CD4 lymphocyte count, 91.7% of physicians indicated they needed at least 1 additional clinical encounter visit before initiating ART for any of these populations (Fig 3). Many physicians (41.2%) indicated that they would need at least 2 additional clinical visits by the patient prior to initiating ART and 20.6% indicated they would need 3 or more visits. These practice patterns do not align with guidelines supporting rapid-start ART (RS-ART) treatment initiation where in the absence of opportunistic infection and patients are ready, there is no contraindication to treatment [2, 28].

## Discussion

While it has been shown in several countries that physicians will withhold ART from some key populations [10–13], to our knowledge, this is the first to investigate physician prescribing strategies in Eastern Europe where HIV incidence and mortality continue to increase [3]. Furthermore, reports from Ukraine in 2019 show extraordinarily low levels of ART coverage in PWH, with only 40% of adult PWH being on ART and even lower coverage for some key populations, especially PWID [29] and prisoners [30]. Thus, the current study sought to investigate physicians as potential barriers to evidence-based HIV treatment in the EECA with a specific focus on Ukraine [31].

### Physician bias and stigma

Stigmas and prejudice underlie the physical act of discrimination and can result in deferment of ART in key populations [17, 32], despite recommendations to the contrary. The current study found that when given the exact same clinical scenarios, physicians may hold certain (implicit) biases towards certain societal groups and are more likely to withhold (i.e., discriminate) ART from these key populations, namely those with substance use disorders (i.e., alcohol and drug use). The decision to defer or withhold ART by physicians based on conscious or unconscious biases poses a unique threat to the control and reduction of HIV around the world, especially in key populations.

HIV stigma world-wide is high, even more so where HIV is criminalized [33]. Stigma, however, is complex where layers of stigma may be synergistic like at the interface of HIV and trans-stigma, homosexuality, or with situations where HIV risk is criminalized as with sex workers and PWID. As a consequence, it has evolved as a syndemic relationship between HIV, stigma, and substance use disorders [34]. In the absence of ART, many individuals within these key populations may experience deteriorated health or worse, continue to engage in HIV risk and in the absence of HIV treatment as prevention, result in onward transmission to others [19]. The antidote to leaving this decision to physicians who may judge their patients is to have a rapid ART initiation irrespective of risk (see below).

### Ongoing alcohol and drug use–High deferral group

Physicians were most likely to defer ART for those with substance use disorders, which is consistent with studies elsewhere [17, 19]. Of considerable interest, and not entirely explained in these findings, is the higher level of ART deferral for those with AUD. Of the WHO-designated high-risk groups mentioned in the study, physicians were most likely to withhold ART in PWH with AUD at all CD4 lymphocyte counts. It is of interest that while Ukraine is a country with one of the highest global annual consumption rates of alcohol [35–37], HIV physicians harbored especially negative attitudes toward such patients [38, 39], and further exploration is warranted. Deferral of ART was almost as high in PWUD and PWID, which might be expected, since drug use, but not alcohol use, is criminalized. When controlling for the feeling thermometer measures toward PWID, stigma in the form of negative stereotypes toward PWID was the major contributor toward ART deferral. While it might be understandable that the criminalization of drug use (PWUD and PWID) as a societal response to drugs may have contributed to high ART deferral levels, the high deferral for those with AUD warrants additional consideration. To better understand this might require delving into the relationships of these physicians with either personal or professional interactions with PWID, PWUD and those with AUD. If this relationship was assessed, it might have been the case in Ukraine that even though alcohol consumption is common and generally tolerated in society [36], personal contact experience may influence bias. Future studies should explore this relationship and assess whether the contact hypothesis may have contributed to their planned action of withholding ART [18, 24, 40].

Ukraine is a prototypical country in the EECA region where HIV is concentrated in PWID with evidence of transmission to their sexual partners. In multivariate correlates examining stereotypes, discrimination intent toward PWID approaches significance and with a larger sample size, we might have observed a difference. Concerning in this finding is that HIV remains heavily concentrated in PWID, who account for 38% of new HIV infections. This proportion, however, is likely much higher as triangulation studies of new HIV cases reported of “sexually transmitted” HIV infections are co-infected with HCV infection, which is likely attributable to drug injection and reflects that injection risk is under-reported [41]. When ART is deferred, it may led to ongoing risk and transmission HIV (and potentially HCV) to others, which is not treatment as prevention [42] and rapid-start ART strategies [2]. Moreover, differential treatment of key populations is divergent from Ukraine’s treatment guidelines that prioritize patients from high-risk groups including those with AUD, PWUD, and PWID.

A number of studies suggest that alcohol and drug use contribute to suboptimal medication adherence [43, 44], though a large meta-analysis suggested that ART adherence levels did not differ in PWID relative to other risk groups [27], but a systematic review suggested that alcohol use disorders negatively influence ART adherence [45]. Despite existing data to the contrary, however, physicians may assume suboptimal ART adherence for PWID and PWUD and extend these perceptions towards others, including released prisoners. As HIV transmission is highly efficient in PWID [42], instead of withholding ART, they should be prioritized for it with fewer treatment demands placed on them. Recent studies during the COVID-19 pandemic in Ukraine suggest that decreasing clinical treatment demands for OAT patients, by allowing less clinical interactions by allowing take-home dosing, improves addiction treatment outcomes [46]. Similarly, a study of clients at syringe services programs achieved high cure rates (94.5%) for HCV by eliminating in-person visits and minimizing diagnostic testing [47].

### Initiation of same-day ART

Physicians poorly predict medication adherence, being able to predict it accurately only half of the time. One potential strategy to avoid differential treatment for some key populations of PWH is to remove physician perception of adherence and move toward rapid ART initiation at the time of diagnosis for all PWH, irrespective of risk behaviors. Studies from Sub-Saharan Africa and Thailand suggest that such strategies are highly effective and improve health outcomes [48–50] in patients who acquire HIV heterosexually; with benefits over standard care confirmed in a Cochrane Review [28]. This study from Ukraine, however, found over half of physicians required at least two or more visits before initiating ART. For patients with lifestyles that do not conform to acceptable norms for physicians, like PWID and PWUD as observed using feeling thermometers, multiple return visits place inordinate demands on patients and reduces likelihood that they will get treated. Demand characteristics have been associated with markedly decreased likelihood of patients receiving evidence-based treatments [51]. Further, even if physicians do not exhibit these biases, such patients may have had negative previous interactions with physicians and they anticipate being treated poorly, further complicating the relationship [52]. Patients want to feel that their physician cares, however, in the settings of high demand characteristics, stigma, and assumed treatment failure for patients with substance use disorders, these pressures become insurmountable and they are lost to follow-up. Rapid or same-day ART initiation may aid in early commitment, appease physicians’ apprehension of treatment non-adherence, and heighten the likelihood of improved outcomes. Such studies of same-day ART, however, are lacking in settings where HIV is concentrated in PWID.

### Substance use disorder mitigation

Being prescribed methadone in PWID appears to reduce the likelihood that physicians would defer ART, a finding that has been previously reported elsewhere [17, 19]. PWID prescribed OAT is associated with improvements along the entire HIV treatment cascade [53], including in Ukraine [54]. These results further suggest that concerns about prescribing ART may be due to concerns about stability and compliance, which has been shown to be mitigated with OAT as it is associated with improved retention in care [54–56]. This is particularly important in understanding the value of OAT in HIV treatment cascades as countries seek to meet the United Nations AIDS 95-95-95 targets [55]. Countries like Ukraine and throughout EECA that have high large numbers of PWID with high HIV prevalence [57], must balance rapid start ART implementation that potentially combine treatment for HIV and opioid use disorder using OAT.

Congruently, prior studies have shown with social support, similar levels of adherence to ART occur among PWID and non-PWID and this has led to adjusted international guidelines regarding prescribing ART to PWID [58, 59]. This further suggests that there have been incorrect assumptions of at risk populations, particularly PWID. For such populations, even if physicians have concerns about adherence, the strategy would be to initiate and provide adherence support rather than withhold ART.

### CD4 count thresholds

It is noteworthy that physicians would increasingly withhold ART as CD4 lymphocyte counts decrease. From a purely clinical perspective, patients with lower CD4 counts are at increased risk for opportunistic infections, non-communicable diseases and death [60, 61]. As Ukraine adopted ART for all PWH irrespective of CD4 count, PEPFAR introduced considerable international funding to support these guidelines to meet 90-90-90 targets. Despite heightened funding for treatment in certain regions, our analysis found that ART deferral did not differ between PEPFAR and non-PEPFAR regions, suggesting that financial support was insufficient to overcome stigma related factors.

One possible explanation for physicians deferring ART at lower CD4 thresholds is their perceived risk that their patient will develop immune reconstitution inflammatory syndrome (IRIS), especially in a country where tuberculosis is so prevalent. While IRIS may happen in some patients, empirical studies from Africa with similar TB rates suggest that ART can still be safely initiated and rapid ART initiation has been successful and safe when patients are briefly screened for symptoms of opportunistic infections [49].

### Sexual and gender minorities

Interestingly, despite low levels of social tolerance in Ukraine toward MSM and transgender women [62–64], withholding ART from these patients did not differ substantially from our “control” patient who contracted HIV through a heterosexual encounter. This finding is consistent with actual ART prescribing practices in Ukraine where ART coverage still remains low, but slightly higher than average for MSM (46% vs 40%) [29]. This finding is similar to reports elsewhere [19]. While further exploration is needed to understand why this was observed, one potential explanation is that physicians providing HIV care have less stereotypically negatively interactions with MSM and TGW, who may have adhered to stringent demands like returning for multiple visits in order to “prove” their interest in treatment. While data for sexual minorities is not available, data from Ukraine suggest that physicians treat PWID poorly in clinical care settings, resulting in PWID discontinuing recommended treatment [65]. Further examination of the contact hypothesis is warranted to disentangle these findings.

Similarly, HIV physicians were no more likely to withhold ART for female sex workers (FSW) relative to our control, even though this behavior is common but criminalized in Ukraine. It is unclear to what extent sex work is distinguished from substance use (which is likely here given the high deferral rate for prisoners) or if treatment as prevention strategies prevailed for this key population. Irrespective of the reason, this bodes well for future HIV prevention in women who sell sex but diverges from findings that ART coverage was low (29%) for sex workers in 2019, much lower than for the population average of 40% [29].

A potential public health solution to reducing stigma and discrimination toward any key population would be to implement RS-ART for all PWH. RS-ART appears to be consistent with a behavioral design intervention (BDI), which aim to influence behavior by presenting choices in a way that guides individuals towards specific decisions [66]. BDIs like RS-ART employ a values-based approach [67, 68] that promotes ethical behaviors and attitudes within social contexts. BDIs use a range of theories, guidelines, and tools to encourage pro-social actions [69, 70] and in the case of RS-ART, it would focus on starting ART not on physician perceptions, but on stated clinical criteria that reduce clinical harms (e.g., presence of opportunistic infections) to patients. Restructuring medical decision-making to overcome stereotype judgment is a hallmark of patient-centered care and aligned with test and treat strategies. Implementing RS-ART studies should be explored as stigma reduction strategies.

### Limitations

Though this study provides several key findings to help guide ART delivery in Ukraine and elsewhere where ART coverage is low, there are some limitations. First, these findings represent treatment intention within hypothetical cases, which could diverge from real-world treatment. Findings here, however, support actual ART coverage in some key populations in the country, which might differ if HIV risk were accurately collected. Second, though providers were recruited from across the country, a convenience sample may not be fully generalizable–though the demographics are similar to HIV treaters in Ukraine who generally very experienced and are mostly women. Third, our results are based on self-report and while social desirability bias may have been present, this was minimized through the survey platform of an anonymous, online survey. Notwithstanding these limitations, the results of this study show that current prescribing practices of ART in Ukraine are not fully in line with the nation’s treatment guidelines, especially with regard to certain key populations and patients with low CD4 counts. Consequently, a divergent policy change that transitions to early treatment initiation for all patients plus combining ART treatment with OAT for PWID with opioid use disorder may overcome some existing barriers.

## Conclusions

Patients with HIV in Ukraine are potentially subject to physician bias toward them, which may in part result in poor individual and public health outcomes. Ukraine relies heavily on international donors for HIV prevention and treatment and given the finding that extra PEPFAR funding had no influence on physicians willing to withhold treatment to some key populations than others, prioritizing funding to promote RS-ART based on clinical criteria first, and then allowing clinical teams to address other factors, including simultaneous RS-ART and RS-OAT, would align with public health mandates.

## Supporting information

S1 Fig Political map of Ukraine outlining the different states (oblasts) across the country*. * The region of Crimea and Sevastopol (in black) is a politically distinct state annexed by Russia and is no longer under the Ministry of Health jurisdiction and thus not included in this study.
(TIF)

S1 Table Percent of HIV treatment providers who would defer antiretroviral therapy for various key populations by CD4+. PWUD = people who use drugs; PWID = people who inject drugs; OAT = opioid agonist therapy; MSM = men who have sex with men. *indicates significance p<0.05 relative to referent (Has an HIV negative sexual partner)–(if p values are <05, .01 and .001), should change the symbols.
(TIF)

S1 File (XLS)
