# Supplementary figures and images for: High rates of deferring antiretroviral treatment for patients with HIV and substance use disorders: Results from a national sample of HIV physicians in Ukraine
Source: PLoS One. 2024 Jul 19;19(7):e0305086. doi: 10.1371/journal.pone.0305086 (PMC11259278; doi:10.1371/journal.pone.0305086)

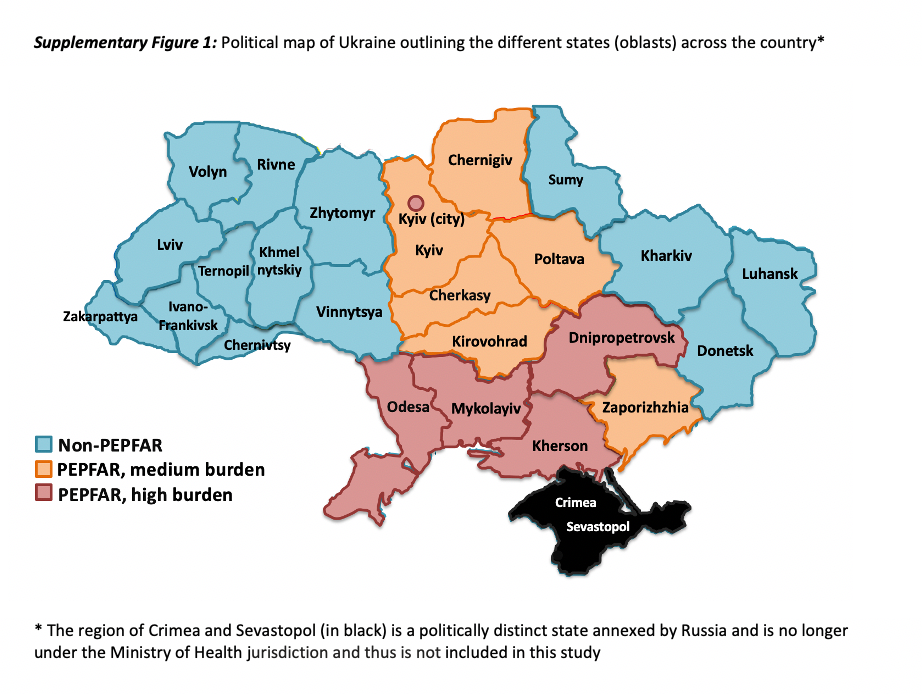

Supplement: S1 Fig — * The region of Crimea and Sevastopol (in black) is a politically distinct state annexed by Russia and is no longer under the Ministry of Health jurisdiction and thus not included in this study. (TIF) [file pone.0305086.s001.tif]

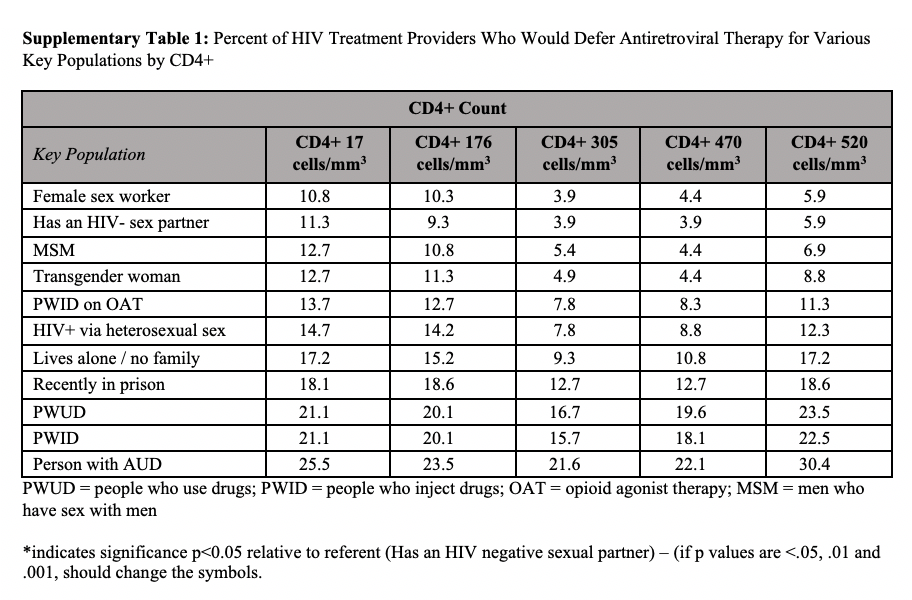

Supplement: S1 Table — PWUD = people who use drugs; PWID = people who inject drugs; OAT = opioid agonist therapy; MSM = men who have sex with men. *indicates significance p<0.05 relative to referent (Has an HIV negative sexual partner)–(if p values are <05, .01 and .001), should change the symbols. (TIF) [file pone.0305086.s002.tif]
